# Artificial temperature-compensated biological clock using temperature-sensitive Belousov–Zhabotinsky gels

**DOI:** 10.1038/s41598-022-27014-z

**Published:** 2022-12-27

**Authors:** Yuhei Yamada, Hiroshi Ito, Shingo Maeda

**Affiliations:** 1 Living Systems Materialogy Research Group, International Research Frontiers Initiative, Tokyo Institute of Technology, 4259, Nagatsuta-Cho, Midori-Ku, Yokohama, 226-8501 Japan; 2 Faculty of Design, Kyushu University, 4-9-1 Shiobaru Minami-Ku, Fukuoka, 815-8540 Japan; 3 Department of Mechanical Engineering, Tokyo Institute of Technology, 2-12-1 Ookayama Meguro-Ku, Tokyo, 152-8550 Japan

**Keywords:** Circadian rhythms, Statistical physics, thermodynamics and nonlinear dynamics

## Abstract

The circadian rhythm is a fundamental physiological function for a wide range of organisms. The molecular machinery for generating rhythms has been elucidated over the last few decades. Nevertheless, the mechanism for temperature compensation of the oscillation period, which is a prominent property of the circadian rhythm, is still controversial. In this study, we propose a new mechanism through a chemically synthetic approach (i.e., we realized temperature compensation by the Belousov–Zhabotinsky (BZ) gels). The BZ gels are prepared by embedding a metal catalyst of the BZ reaction into the gel polymer. We made the body of BZ gels using a temperature-sensitive polymer gel, which enabled temperature compensation of the oscillation by using temperature dependence of volume. Moreover, we constructed a simple mathematical model for the BZ oscillation in temperature-sensitive gels. The model can reproduce temperature compensation of BZ gels, even though all reactions are temperature sensitive according to the Arrhenius rule. Our finding hints that a soft body coupling may be underlying temperature-compensated biological functions, including circadian rhythms.

## Introduction

Circadian rhythm is a biological phenomenon, which repeats with a period of about 24 hours^1^. Almost all organisms, from bacteria to mammals, exhibit circadian rhythms. Examples include the sleep–wake cycle in humans and the nyctinastic movement of leaves in plants^2^. The molecular mechanisms for producing circadian rhythms depend on the domain of the organism. That is, the genes responsible for circadian rhythms in animals, plants, and bacteria do not overlap.

However, pioneering circadian studies found that almost all organisms have three common properties. The first property is that circadian rhythms persist even under constant environmental conditions, implying that an endogenous self-sustained oscillator generates the circadian rhythms. The second is the synchronization of the circadian rhythms with diurnal light and temperature variations. According to the theory of synchronization, cyclic external forces applied to a self-sustained oscillator always result in synchronization when the force is sufficiently strong and the period of the external force is similar to that of the oscillator^3^. Therefore, if the oscillator is light or temperature sensitive, the second property follows from the first without any special assumptions. The third property is that the oscillation period does not significantly change under different temperature, even though the rates of most biochemical reactions are temperature-sensitive, which is called the temperature compensation. This property allows organisms to have a precisely ticking clock, regardless of the season and consequently synchronize with environmental changes with a 24-h period.

The reconstruction by chemistry is one of the tools to elucidate circadian rhythms. In particular, the first property, self-sustained rhythms, has been well studied through chemical oscillations. One of the well-known chemical oscillators is the Belousov–Zhabotinsky (BZ) reaction. The BZ reaction in the 1960s gave rise to its position as a typical chemical oscillator^4^. Additionally, some artificial oscillators, including the BZ reaction, are synchronizable to environmental cycles. For example, the BZ reaction can be synchronized to light cycles^5–7^, meaning that the second property of circadian rhythm has been artificially reconstructed. Moreover, developing mathematical models for these artificial oscillators is easy compared with a natural biological oscillator because the molecules that produce the artificial oscillations are clear.

Chemical reconstruction of the third property requires a special mechanism, since the rate constants of chemical reactions follow the Arrhenius law, where the rate constants vary exponentially with temperature. The BZ reaction is no exception^8^. $Q_{10}$, which is a common measure for the temperature compensation in circadian research, indicates how many times faster the rate constant becomes when the environmental temperature changes by 10 °C. In a study, it is calculated for the BZ reaction experiment in a stirred solution system as $Q_{10} \approx 2$ in the range 40–80 °C^9^. Generally, most biochemical reactions are also temperature dependent and show $Q_{10} \approx$ 2–3^10^, although $Q_{10}$ in real circadian systems ranges between 0.8 and 1.2^2^.

A possible hypothesis for the temperature compensation mechanism is that a network of biochemical reactions is exquisitely designed. Namely, even if raising the temperature increases a reaction rate that determines the period of oscillation, another temperature-sensitive inhibitory molecule simultaneously slows the period-determining reaction, resulting in the balance of period^11–13^. There are both experimental and theoretical studies on the mechanism that utilizes chemical interactions of proteins^14,15^, temperature-amplitude coupling^16^, reaction networks with feedback loop^17^, and so on^18,19^. Focusing on chemical oscillators, including the BZ reaction, the period becomes temperature compensated when the values of activation energy suffice a certain balance equation^20^. Some studies have experimentally demonstrated that the mechanism actually works^21–23^. In the case of BZ reactions, it is reported that substrate composition and initial reagent concentrations effect on the activation energy and when they are well controlled, ${Q}_{10}=1.3$ can be accomplished^24^.

In the previous studies, the temperature compensation mechanism often required careful adjustment of chemicals. This paper proposes and implements a different idea; we present a new temperature-compensation mechanism that uses thermo-sensitivity of gels. Polymer gels, which are mostly composed of polymer chains and a solvent, are quality places to induce chemical reactions. In particular, stimuli-sensitive gels undergo volume changes in response to environmental changes. Examples include thermo-sensitive gels exhibiting significant volume changes in response to changes in temperature^25,26^.

**Figure 1:**
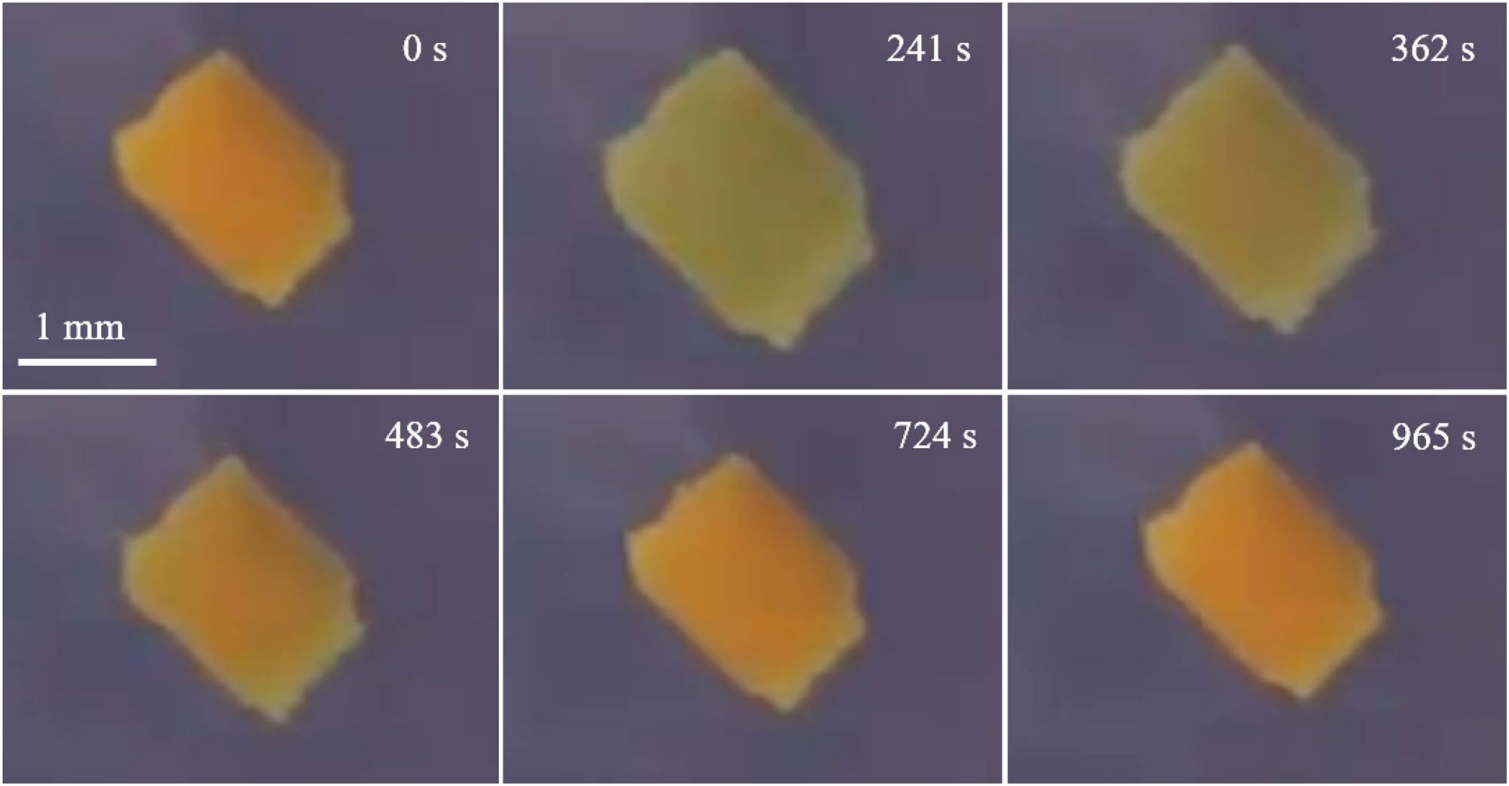
Snapshots of the BZ gel’s oscillation. Top right shows the time stamp.

We have developed active gels that synchronize with the BZ reaction and generate volume oscillations on a macroscopic scale^27–29^ In 1996, BZ gels were reported to exhibit a slight volume oscillation^30^. The BZ reaction is an oxidation reaction of organic acids under acidic conditions, and the valence of various reaction intermediates and metal ions oscillate during the reaction process. The BZ reaction shows a typical limit cycle oscillation. The concentration of a chemical substance oscillates in a stirred state, and the chemical wave propagates in an unstirred state. Since the BZ reaction obeys the Arrhenius equation^9^, usual BZ solution system does not show temperature compensation unless the compound of chemicals is carefully chosen^9,10,24^. When the BZ reaction occurs within a polymer gel to which the metal complex of the BZ reaction is covalently bound, the gel undergoes a periodic volume oscillation and peristaltic motion. When BZ gels smaller than the wavelength of the chemical wave are prepared, redox changes in the BZ gel occur homogeneously without stirring the solution (Fig. 1).

**Figure 2:**
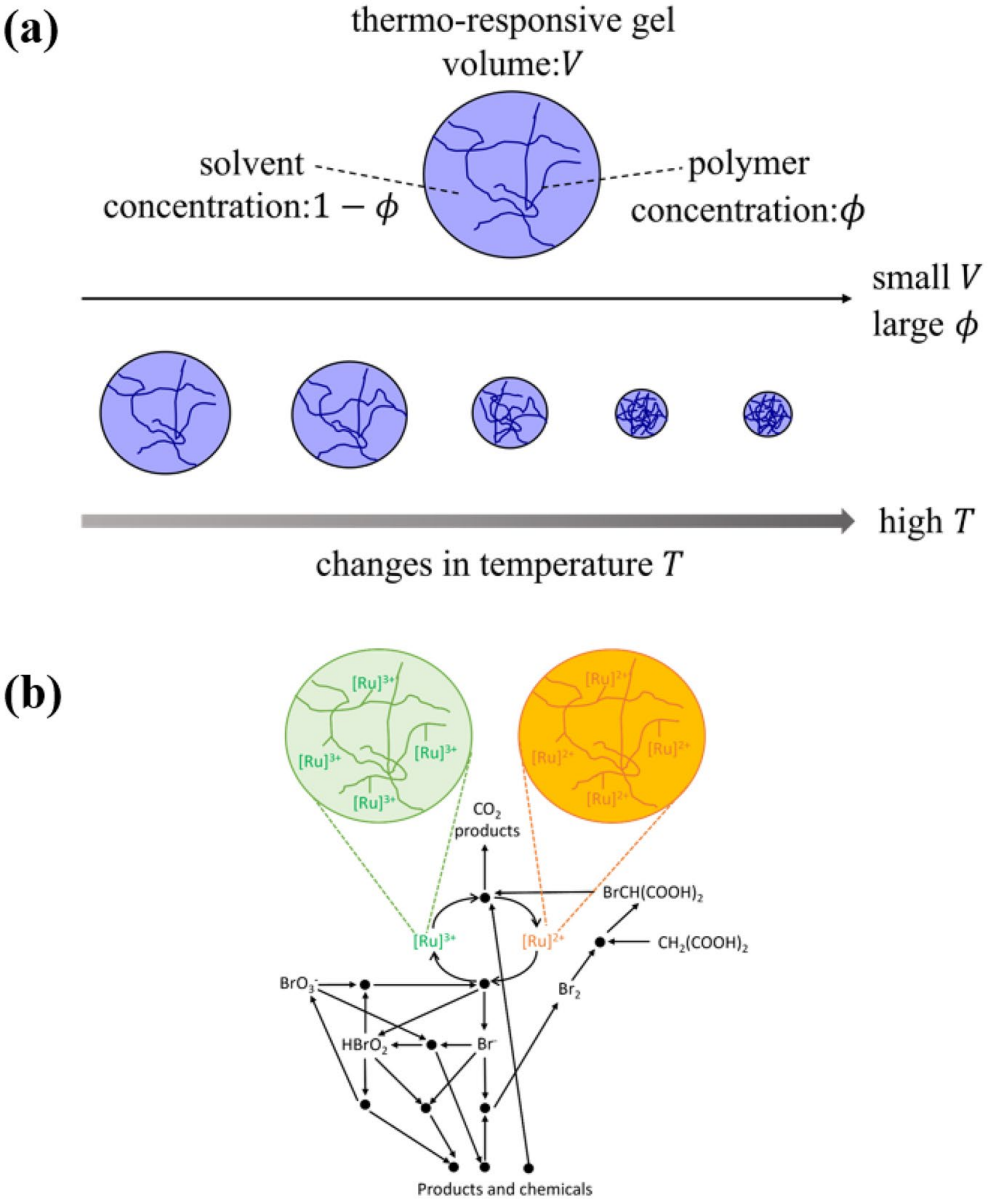
Schematic image of the hermos-responsive BZ gels. (**a**) Temperature dependence of the gel volume and volume fraction. (**b**) Chemical reaction network of the BZ reaction.

In this study, we used poly (N-isopropylacrylamide) (PNIPAAm), which is temperature-responsive, to synthesize temperature-compensating BZ gels. PNIPAAm gels change their volume in response to temperature changes (Fig. 2). In other words, the concentration $\phi$ of the polymer in the gel and the solvent concentration $1-\phi$ change with changes in temperature. At higher temperatures, PNIPAAm gels contract and exhibit higher $\phi$. At lower temperatures, PNIPAAm gels expand and exhibit lower $\phi$. By using the temperature responsiveness of the PNIPAAm gels, the concentration of the BZ reaction induced in the BZ gels can be controlled.

Here, we propose using the temperature responsiveness of PNIPAAm gels to realize the function of temperature compensation in the BZ gels. Thus, as the temperature changes, the polymer concentration in the BZ gel changes and automatically controls the period of the BZ reaction. As a result, the BZ gel exhibits temperature compensatory properties. We constructed a simple mathematical model for BZ gels to show temperature compensability, which indicates that a BZ gel exhibits temperature compensability like circadian rhythm observed in living organisms. Then we synthesized temperature-compensating BZ gels based on mathematical analysis and experimentally demonstrated thehermosuree-compensating property of BZ gels.

## Model and analysis

A previous study proposed a model of BZ gel dynamics^31^. Based on Tyson and Fife’s version of the Oregonator, they modeled the BZ reaction in a gel system via the following chemical reactions:

$$\left( {1 - \phi } \right)A + Y\mathop{\longrightarrow}\limits^{{k_{1} \left( {1 - \phi } \right)^{2} H^{2} }}X + P$$

$$X + Y\mathop{\longrightarrow}\limits^{{k_{2} \left( {1 - \phi } \right)H}}2P$$

$$\left( {1 - \phi } \right)A + X\mathop{\longrightarrow}\limits^{{k_{3} \left( {1 - \phi } \right)H}} 2X + 2Z$$

$$X + X\mathop{\longrightarrow}\limits^{{k_{4} }}\left( {1 - \phi } \right)A + P$$

$$\left( {1 - \phi } \right)B + Z\mathop{\longrightarrow}\limits^{{k_{5} }}\frac{1}{2}fY$$

where $A = [{\text{BrO}}_{3}^{ - } \left] {, H = } \right[{\text{H}}^{ + } \left] {,P = \left[ {{\text{HOBr}}} \right],B = } \right[{\text{BrCH}}\left( {{\text{COOH}})_{2} \left] {,X = } \right[{\text{HBrO}}_{2} \left] {,Y = } \right[{\text{Br}}^{ - } \left] {,Z = } \right[{\text{M}}_{{{\text{OX}}}} } \right]$ (oxidized metal catalyst), $f$ is a stoichiometric factor, and $\phi$ is the volume fraction of the polymer gel. Focusing on the dynamics of $X,Y$ and $Z$, the concentration variation due to swelling and deswelling as well as the chemical reaction should be considered. Denoting $\tau$ as time and applying the approximation $dY/d\tau \simeq 0$ (which is often assumed for the BZ reaction), they obtained the following nondimensionalized differential equations

1 $$\frac{du}{{dt}} = - \frac{u}{1 - \phi }\frac{d\phi }{{dt}} + F\left( {u,v,\phi } \right)$$

2 $$\frac{dv}{{dt}} = \frac{v}{\phi }\frac{d\phi }{{dt}} + \epsilon{G\left( {u,v,\phi } \right)}$$

3 $$F\left( {u,v,\phi } \right) = \left( {1 - \phi } \right)^{2} u - u^{2} - fv\left( {1 - \phi } \right)\frac{{u - q\left( {1 - \phi } \right)^{2} }}{{u + q\left( {1 - \phi } \right)^{2} }}$$

4 $$G\left( {u,v,\phi } \right) = \left( {1 - \phi } \right)^{2} u - \left( {1 - \phi } \right)v$$

In these equations, nondimensional variables $u,v$, and $t$ are defined as

5 $$u = \frac{X}{{X_{0} }}, v = \frac{Z}{{Z_{0} }}, t = \frac{\tau }{{\tau_{0} }}$$

6 $$X_{0} = \frac{{k_{3} HA}}{{2k_{4} }}, Z_{0} = \frac{{\left( {k_{3} HA} \right)^{2} }}{{k_{4} k_{5} B}}, \tau_{0} = \frac{1}{{k_{3} HA}}$$

7 $$\epsilon = \frac{{k_{5} B}}{{k_{3} HA}}, q = \frac{{2k_{1} k_{4} }}{{k_{2} k_{3} }}$$

To describe the BZ gel dynamics, we also need an equation for $\phi$. In actual systems, $\phi$ is determined by balancing the osmotic pressure, which depends on $v$ and the elastic stress. However, it is often reported that the variation in the gel volume induced by oscillation of $v$ is not very large in the experiments. Below, we ignore the variation for simplicity. Assuming $\phi$ is constant, Eqs. (1) and (2) become

8 $$\frac{du}{{dt}} = F\left( {u,v,\phi } \right)$$

9 $$\frac{dv}{{dt}} = \epsilon{G\left( {u,v,\phi } \right)}$$

Mathematically, these equations restore the two-variable Oregonator model when $\phi = 0$.

In this model, the temperature affects the reaction rate constants of each chemical reaction. Assuming $k_{j} \left( {j = 1,2, \ldots ,5} \right)$ obeys the Arrhenius law, then

10 $$k_{j} = k_{j}^{*} {\text{exp}} \left[ { - \frac{{E_{j} }}{R}\left( {\frac{1}{T} - \frac{1}{{T_{0} }}} \right)} \right]$$

where $T$ is the absolute temperature, $T_{0}$ is room temperature, $R$ is the universal gas constant, $E_{j}$ is the activation energy, and $k_{j}^{*}$ is the reaction rate constant at $T = T_{0}$. Then the temperature dependences of $\epsilon$ and $q$ become

11 $$\epsilon = \epsilon_{0} {\text{exp}} \left[ { - \frac{{E_{3} - E_{5} }}{R}\left( {\frac{1}{T} - \frac{1}{{T_{0} }}} \right)} \right]$$

12 $$q = q_{0} {\text{exp }}\left[ { - \frac{{E_{2} + E_{3} - E_{1} - E_{4} }}{R}\left( {\frac{1}{T} - \frac{1}{{T_{0} }}} \right)} \right]$$

where

13 $$\epsilon_{0} = \frac{{k_{5}^{*} B}}{{k_{3}^{*} HA}}, q_{0} = \frac{{2k_{1}^{*} k_{4}^{*} }}{{k_{2}^{*} k_{3}^{*} }}$$

Since $t$ is a dimensionless variable normalized by the rate constants, the dimension must be restored to consider the period in real time. When the oscillation period in $t$ is $\Delta t$, the period in real time, $\Delta \tau$, becomes

14 $$\Delta \tau = \tau_{0} \Delta t = {\text{exp}} \left[ {\frac{{E_{3} }}{R}\left( {\frac{1}{T} - \frac{1}{{T_{0} }}} \right)} \right] \Delta t$$

Note that this discussion is almost parallel to the discussion of a temperature-dependent Oregonator model^32^. As in the literature, one can numerically compute the detailed behavior of this model, though, here we proceed an analytical calculation to reveal an underlying mathematical structure by adopting some approximations. Following the work on the Oregonator’s oscillation period^33^, the oscillation period of our model is estimated as

15 $$\Delta t \simeq \frac{1}{{ \epsilon \left( {1 - \phi } \right)}}{\text{log}} \left[ {\frac{1}{{4q\left( {1 - q} \right)}}} \right]$$

(Details of the analysis are presented in the Supplementary Information.) Since $q \ll 1$, we approximate $1 - q$ in log as $1$. From Eq. (14), the temperature dependency of $\Delta \tau$ other than $\phi$ becomes

16 $$\Delta \tau = \frac{1}{1 - \phi }\frac{1}{{ \epsilon_{0} k_{3}^{*} HA}}e^{{\frac{{E_{3} }}{R}\left( {\frac{1}{T} - \frac{1}{{T_{0} }}} \right)}} \left[ {\frac{{E_{1} + E_{4} - E_{2} - E_{3} }}{R}\left( {\frac{1}{T} - \frac{1}{{T_{0} }}} \right) - {\text{log}}4q_{0} } \right]$$

Here, the latter bracket [] is approximated as a constant $\alpha$ in the variation of $T$ at room temperature ± 10 °C when the typical parameter values for the BZ reaction are substituted. Then

17 $$\Delta \tau \simeq \alpha \frac{1}{1 - \phi }\frac{1}{{k_{5}^{*} B}}e^{{\frac{{E_{5} }}{R}\left( {\frac{1}{T} - \frac{1}{{T_{0} }}} \right)}}$$

**Figure 3:**
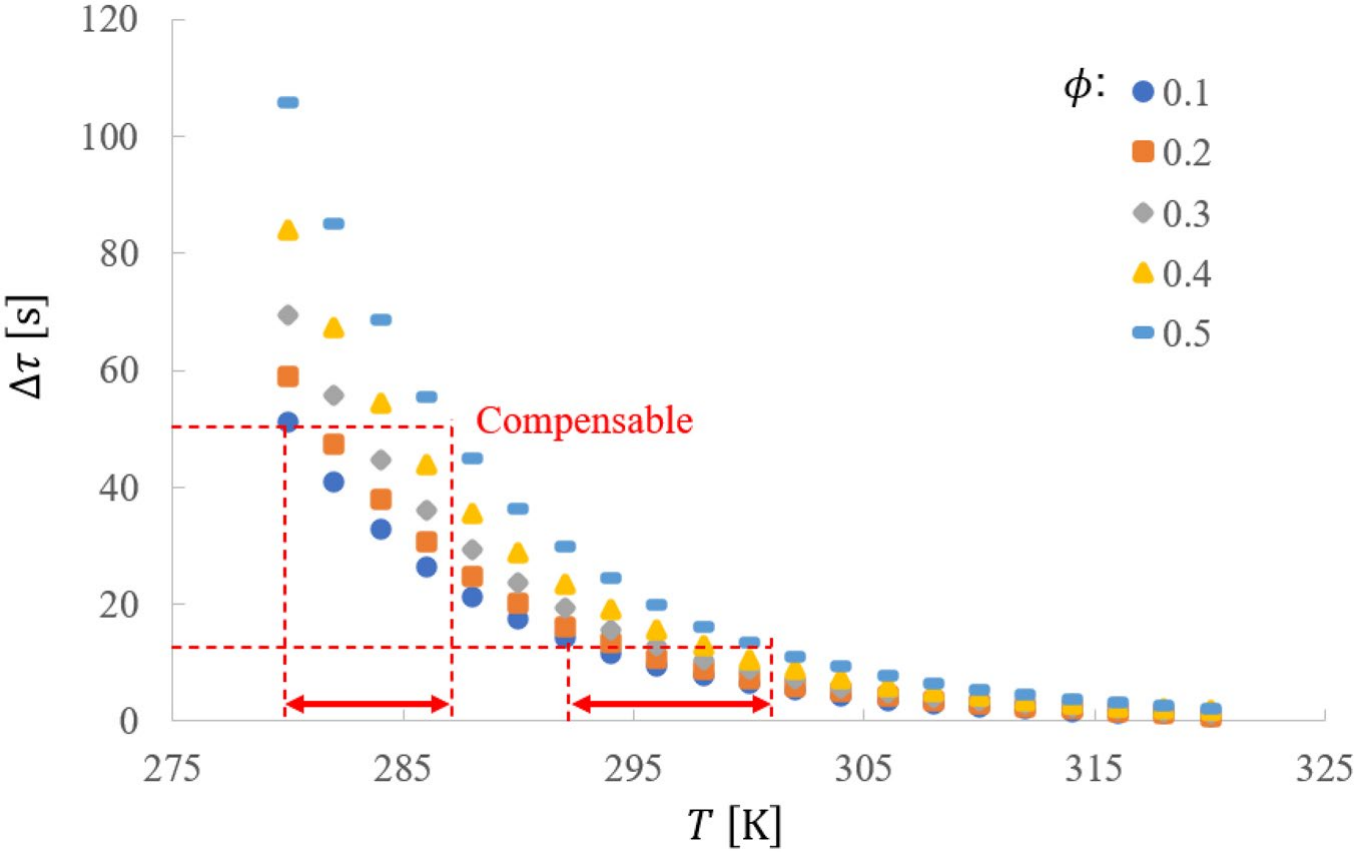
A theoretical estimation of the BZ gel’s oscillation period ${\Delta }\tau$ with varying temperature $T$ and volume fraction $\phi$, which is given by Eq. (16). Here, rate constants and activation energies at $T = T_{0} = 298 \left[ {\text{K}} \right]$ are set as $k_{1}^{*} = 2 \left[ {{\text{mol}}^{ - 3} {\text{s}}^{ - 1} } \right], k_{2}^{*} = 10^{6} \left[ {{\text{mol}}^{ - 2} {\text{s}}^{ - 1} } \right], k_{3}^{*} = 10 \left[ {{\text{mol}}^{ - 2} {\text{s}}^{ - 1} } \right], k_{4}^{*} = 2000 \left[ {{\text{mol}}^{ - 1} {\text{s}}^{ - 1} } \right], k_{5}^{*} = 1 \left[ {{\text{mol}}^{ - 1} {\text{s}}^{ - 1} } \right], E_{1} = 54 \left[ {{\text{kJ mol}}^{ - 1} } \right], E_{2} = 25 \left[ {{\text{kJ mol}}^{ - 1} } \right], E_{3} = 60 \left[ {{\text{kJ mol}}^{ - 1} } \right], E_{4} = 64 \left[ {{\text{kJ mol}}^{ - 1} } \right], E_{5} = 70 \left[ {{\text{kJ mol}}^{ - 1} } \right]$^32^. Temperature compensable ranges are depicted.

Figure 3 shows the theoretical estimation of the oscillation period with varying $T$ and $\phi$. This relation suggests that as the temperature increases, the decrease in the period is suppressed when $\phi$ increases. When we choose an appropriate polymer whose $\phi$ varies with $T$ as maintaining ${\Delta }\tau$ at a constant, we can realize temperature compensated oscillation of the BZ gel.

## Results and discussion

**Figure 4:**
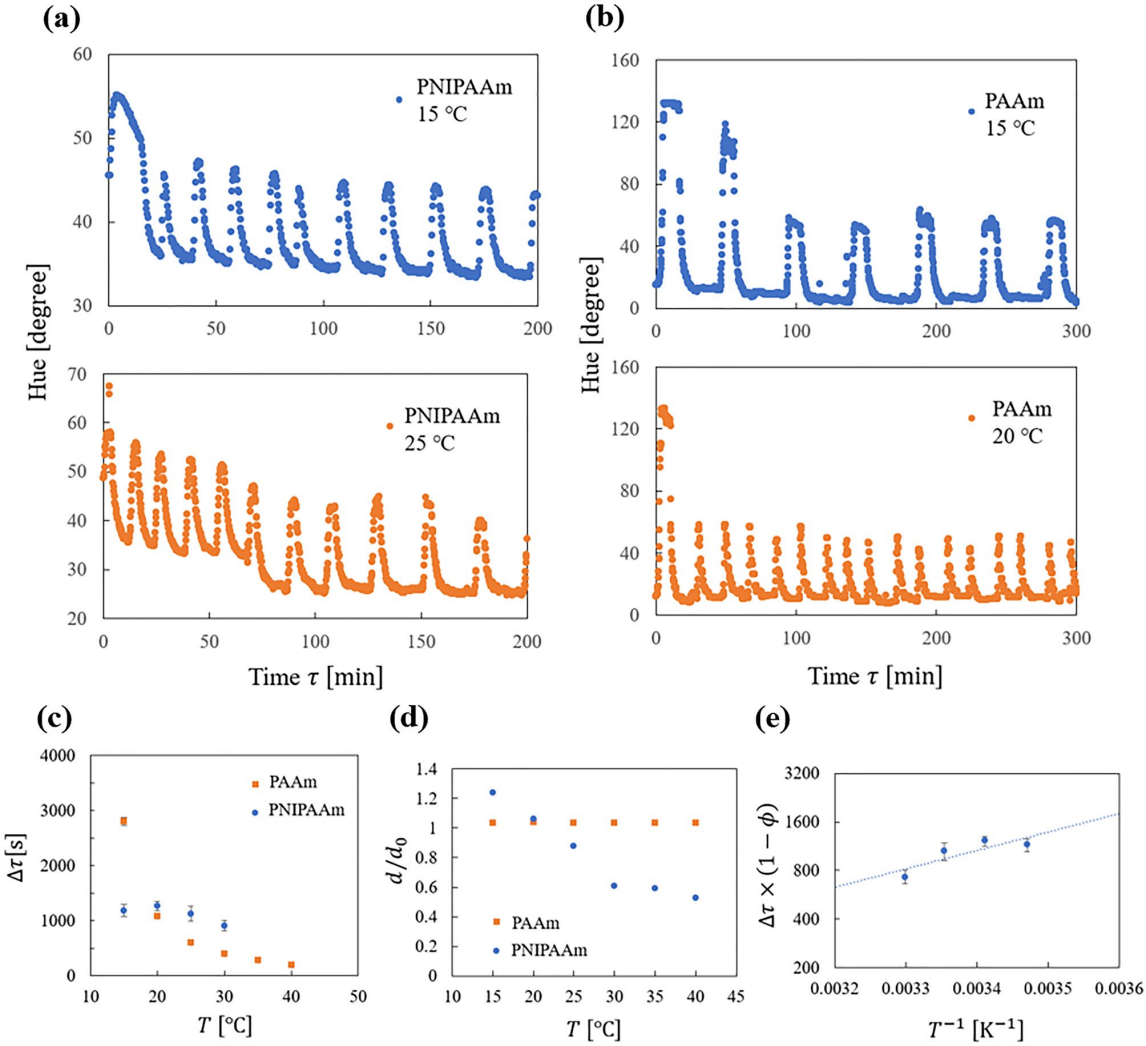
(**a**) Typical time evolution of hue for BZ gels obtained for PNIPAAm, 15 °C, 25 °C. (**b**) That for PAAm, 15 °C, 20 °C. (**c**) Temperature dependence of the period. Orange square and blue circle show the case for PAAm and PNIPAAm, respectively. Error bars are the standard error over the average of 5 samples for PAAm 15 °C and 10 samples for the others. (Error bars are omitted when point size is larger than that.) (**d**) Temperature dependence othe BZ gel diameter. The vertical value is divided by the diameter when the gel is synthesized. Orange square and blue circle show the case for PAAm and PNIPAAm, respectively. (**e**) Inverse temperature vs. period times $\left( {1 - \phi } \right)$. Points show experimental data, and the dashed line shows the fitting curve using Eq. (19). Error bars are the same as that of in (**d**).

We synthesized the BZ gels with two types of gel: PNIPAAm and PAAm. The PNIPAAm gel decreases its volume when the temperature increase,s while the PAAm gel maintains a constant volume. (Details of the synthesis and analysis are presented in the Supplementary Information. Examples of the oscillation are given as Supplemental Videos.) We observed the oscillation while varying the temperature. From the experiment, we obtained the time series of BZ gel hue, which oscillates according to the variation in the chemical concentration. Figure 4a and b show typical time series of the hue change. The hue value initially becomes large, which corresponds to the convergence to limit the cycle, followed by a relatively steady oscillation. In the time series, we evaluated the oscillation period as the time interval between two neighboring peaks. Since the period fluctuates with time, we calculated the mean period as the time average over the first ten oscillations after the second peak. Figure 4c shows the temperature dependency of the BZ gel oscillation period. The behavior depends on the type of gel. It is confirmed that the BZ gel composed of PNIPAAm shrinks with increasing the temperature, while that composed of PAAm does not change its volume.

Here, we calculate $Q_{10}$ for the oscillations. $Q_{10}$ is given as

18 $$Q_{10} = \left( {\frac{{\Delta \tau_{1 } }}{{\Delta \tau_{2 } }}} \right)^{{\frac{10}{{T_{2} - T_{1} }}}}$$

where ${\Delta \tau }_{1}$ and ${\Delta \tau }_{2}$ are the oscillation periods of the reaction at temperature ${T}_{1}$ and ${T}_{2}$, respectively. In the range of 15–30 °C, ${Q}_{10}=3.73$ for PAAm and ${Q}_{10}=1.19$ for PNIPAAm. The value for PNIPAAm is within the range of typical circadian rhythms.

Since Eq. (17) suggests

19 $$\Delta \tau \left( {1 - \phi } \right) \sim e^{{\frac{\beta }{T}}}$$

the product of the period and $\left( {1 - \phi } \right)$ must be an exponential function of the inverse temperature if the analysis is valid. Here, we check this relation. Figure 4d shows the temperature dependence of the BZ gel diameter. This clearly shows a different temperature dependence of the two gels’ volume. From that, we calculate the volume ratio as

20 $$\frac{V}{{V_{0} }} = \left( {\frac{d}{{d_{0} }}} \right)^{3}$$

Then we calculated the volume fraction as

21 $$\phi = \frac{{\phi_{0 } V_{0} }}{V}$$

where ${\phi }_{0}=0.045$ is the initial volume fraction, which is calculated assuming it has the same value as the mol fraction when it is synthesized. Figure 4e shows $\Delta \tau (1-\phi )$ versus temperature. The dashed line indicates a fitting curve using Eq. (19). The result is consistent with our analysis.

## Conclusion

This paper suggests a possibility that temperature compensation can be naturally embedded in self-sustainability through the output system of circadian machinery. This linkage may explain why temperature compensation is a universal property of the circadian rhythm, which is valid for animals, plants, and bacteria, regardless of the molecular species.

Some reports seem to support the key assumption of this study is not absurd. It has been reported that some cellular volumes are actually temperature dependent^34^. The cellular volume can even show circadian rhythm similar to that of the BZ gel^35^. Moreover, the amount of clock proteins localized in the nucleus is crucial for circadian rhythms because auto-inhibitory regulation by the protein transported into nucleus generates the rhythms. In plant cells, this nucleus localization depends on temperature, meaning that temperature controls the concentration of the oscillatory components^36^. The localization of clock proteins occurs even in cyanobacteria, whose clock works without transcriptional-translational feedback^37,38^. This localization will contribute to the temperature compensation if it occurs in a temperature-dependent manner.

## Supplementary Information

Supplementary Information 1.

Supplementary Video 1.

Supplementary Video 2.

Supplementary Video 3.

Supplementary Video 4.

## Data Availability

All data generated or analyzed during this study are included in this published article and its supplementary information files.
