# Author Correction: Artificial temperature-compensated biological clock using temperature-sensitive Belousov–Zhabotinsky gels

**DOI:** 10.1038/s41598-023-41310-2

**Published:** 2023-08-29

**Authors:** Yuhei Yamada, Hiroshi Ito, Shingo Maeda

**Affiliations:** 1 Living Systems Materialogy Research Group, International Research Frontiers Initiative, Tokyo Institute of Technology, 4259, Nagatsuta-Cho, Midori-Ku, Yokohama, 226-8501 Japan; 2 Faculty of Design, Kyushu University, 4-9-1 Shiobaru Minami-Ku, Fukuoka, 815-8540 Japan; 3 Department of Mechanical Engineering, Tokyo Institute of Technology, 2-12-1 Ookayama Meguro-Ku, Tokyo, 152-8550 Japan

Correction to: *Scientific Reports* 10.1038/s41598-022-27014-z, published online 27 December 2022

The original version of this Article contained a typographical error in Equations 2 and 9, where ‘$\epsilon$’ was omitted.

**Equation 2:**

$$ \frac{dv}{{dt}} = \frac{v}{\phi }\frac{d\phi }{{dt}} + G\left( {u,v,\phi } \right)\quad (2) $$

now reads:

$$ \frac{dv}{{dt}} = \frac{v}{\phi }\frac{d\phi }{{dt}} + \epsilon G\left( {u,v,\phi } \right)\quad (2) $$

**Equation 9:**

$$ \frac{dv}{{dt}} = G\left( {u,v,\phi } \right)\quad (9) $$

now reads:

$$ \frac{dv}{{dt}} = \epsilon G\left( {u,v,\phi } \right)\quad (9) $$

The original Article has been corrected.

