# Supplementary material for: Artificial temperature-compensated biological clock using temperature-sensitive Belousov–Zhabotinsky gels
Source: Sci Rep. 2022 Dec 27;12:22436. doi: 10.1038/s41598-022-27014-z (PMC9794784; doi:10.1038/s41598-022-27014-z)
Supplement: Supplementary file 1 — Supplementary Information 1. [file 41598_2022_27014_MOESM1_ESM.pdf]

Materials and Methods

Synthesis of BZ gels

For the PNIPAAm BZ gels, we prepared the temperature-sensitive BZ gels in the following way: We added 0.573 g of NIPAAm, 0.00813 g of MBAA as a crosslinker, 0.0712 g of ruthenium(4-vinyl-4-methyl-2,2-bipylridine) bis (2,2-bipyridine) bis (hexafluorophosphate) $[Ru(bpy)_3]^{2+}$) and 0.00866 g of AIBN as an initiator to methanol (1.5 ml). We then stirred the resulting solution for 30 min. We added 0.0182 g of 2-acrylamido-2-methylpropanesulfonic acid (AMPS) to pure water (1.5 ml) and stirred the resulting solution. We mixed two solutions together. We then stirred the mixed solution and purged it with dry nitrogen gas. We injected the mixed solution into glass capillaries with a diameter of 1.0 mm. We put these glass capillaries into an oven at 60 ºC for 20 h. We finally washed the gel samples carefully. The reaction solution of the BZ reaction was prepared by mixing nitric acid, sodium bromate and malonic acid in the ratio of 1:1:3 in the Molar concentration.

For the PAAm BZ gels, which are non-temperature-sensitive, we followed the same procedure except for the quantity of chemicals as following way: We added 0.528 g of AAm, 0.00119 g of MBAA, 0.104 g of $[Ru(bpy)_3]^{2+}$ and 0.00866 g of V-50 as an initiator to methanol (1.5 ml). We then stirred the resulting solution for 30 min. We added 0.0266 g of AMPS to pure water (1.5 ml) and stirred the resulting solution. The subsequent synthesis process for the PAAm BZ gels is similar to that described above.

Observation and data analysis

For the observation, the BZ gels are placed in a constant temperature bath filled with the reaction solution (Supplemental Figure S1). The temperature is controlled by circulating water. We recorded videos of the BZ gels from vertical direction. Supplemental Videos are the examples. S1, S2, S3 and S4 correspond to the cases for PAAm BZ gel at 15°C, PAAm BZ gel at 40°C, PNIPAAm BZ gel at 15°C and PNIPAAm BZ gel at 30°C, respectively. All of them are 256 times speed. To obtain the time series of hue value, we fixed a pixel of the BZ gel in the video and calculated the hue (Fig. 3).

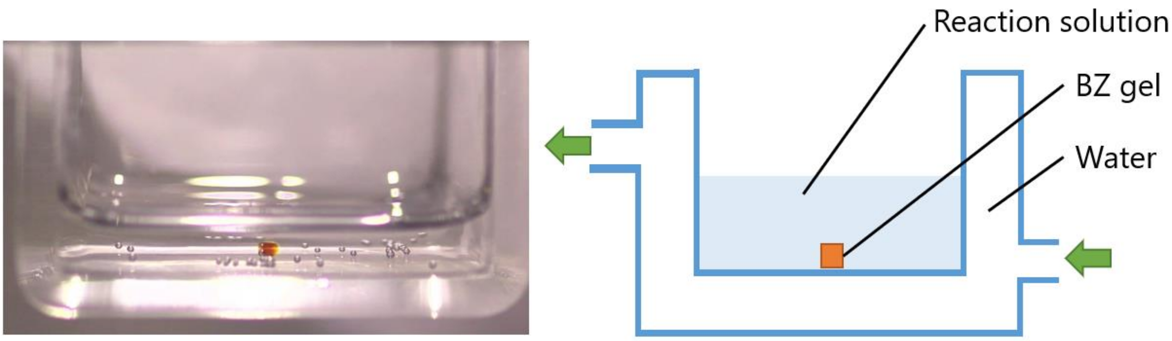

Fig. S1 Schematics of the experimental system.

Supplemental Analysis

Estimation of period

Here, we estimate the oscillation period of the limit cycle. Supplemental Figure S2 shows a numerically obtained trajectory of the limit cycle and nullclines of differential equations (8) and (9) in the main text. Purple points show the trajectory of limit cycle at a certain interval. The trajectory moves counterclockwise as t increases and it stays most of the time in a narrow domain between the cubic nullcline and the v axis. So, the period of the limit cycle is estimated by the time for the trajectory to pass the domain.

The nullclines of differential equations (8) and (9) are

$$v = \frac{1}{f(1-\phi)}\{(1-\phi)^2 u - u^2\}\frac{u + q(1-\phi)^2}{u - q(1-\phi)^2} \qquad \text{(S1)}$$

$$v = (1-\phi)u \qquad \text{(S2)}$$

Now we denote the range of trajectory as C and the time for the state to pass C as Δt_C. Denoting speed of state in phase space as U and the infinitesimal distance along trajectory as dl, Δt_C is given as

$$\Delta t_C = \int_C \frac{1}{V} dl \qquad \text{(S3)}$$

where $V = \sqrt{\dot{u}^2 + \dot{v}^2}$ and $dl = \sqrt{du^2 + dv^2}$. Because the trajectory is almost along with the nullcline (S1) in C, $\dot{u} \simeq 0$. Additionally, because $u \simeq 0$ in C, $\dot{v} \simeq -\epsilon(1-\phi)v$. Then, denoting the maximum and minimum of u in C as $v_{\max}$ and $v_{\min}$, respectively,

$$\Delta t_C \simeq \int_{v_{\min}}^{v_{\max}} \frac{1}{\dot{v}} dv = -\frac{1}{\epsilon(1-\phi)} \int_{v_{\min}}^{v_{\max}} \frac{1}{v} dv \qquad \text{(S4)}$$

$v_{\max}$ and $v_{\min}$ can be estimated approximately as follows. As shown in Fig. S1, $v_{\max}$ and $v_{\min}$ are almost the same as the maximal and minimal value of the nullcline (Fig. S2). Near the maximal point, since $u \gg q(1-\phi)^2$, Eq. (S1) is approximated as

$$v \simeq \frac{1}{f(1-\phi)} \{(1-\phi)^2 u - u^2\}$$

$$= -\frac{1}{f(1-\phi)} \left[\left\{ u - \frac{(1-\phi)^2}{2} \right\}^2 - \frac{(1-\phi)^4}{4} \right] \qquad \text{(S5)}$$

Then, we obtain

$$v_{\max} \simeq \frac{(1-\phi)^3}{4f} \qquad \text{(S6)}$$

The minimal point of nullcline (S1) emerges at $u \simeq q(1-\phi)^2$. Thus, we can estimate the minimal value by substituting it into Eq. (S5), which becomes

$$v_{\min} \simeq \frac{(1-\phi)^3}{f} q(1-q) \qquad \text{(S7)}$$

Then, substituting Eqs. (S6) and (S7) into Eq. (S4), we obtain

$$\Delta t_C \simeq \frac{1}{\epsilon(1-\phi)} \log \left[\frac{1}{4q(1-q)} \right] \qquad \text{(S8)}$$

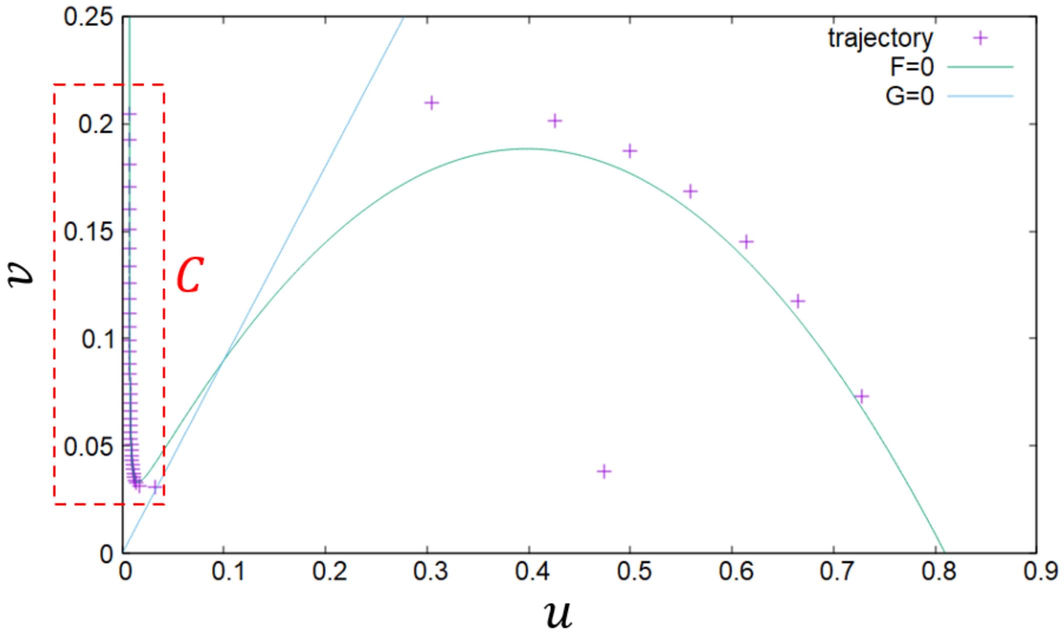

Fig. S2 Nullclines and trajectory of our model. $f = 1.0, q = 0.008, \epsilon = 0.1, \phi = 0.1$ are adopted as typical parameters. Green curve and blue line show nullclines Eq. (S1) and Eq. (S2), respectively. Purple dots show limit cycle plotted in time interval $\Delta t = 7$, which is obtained by numerically integrating differential equations (8) and (9).
